# Invasive *Malassezia pachydermatis* Infection in an 8-Year-Old Child on Lipid Parenteral Nutrition

**DOI:** 10.1155/2022/8636582

**Published:** 2022-01-20

**Authors:** Zheyi Teoh, Joel Mortensen, Joshua K. Schaffzin

**Affiliations:** ^1^Division of Pediatric Infectious Diseases, Cincinnati Children's Hospital Medical Center, Cincinnati, OH, USA; ^2^Department of Pathology and Laboratory Medicine, Cincinnati Children's Hospital Medical Center, Cincinnati, OH, USA; ^3^Department of Pediatrics, University of Cincinnati College of Medicine, Cincinnati, OH, USA

## Abstract

Invasive disease due to *Malassezia pachydermatis* infection is uncommon but increasingly recognized in children, especially neonates on parenteral nutrition or immunocompromised children. We describe a case of *Malassezia pachydermatis* fungemia in a demographically distinct patient and discuss the workup and current strategies for managing this infection in the setting of a central venous catheter.

## 1. Introduction

The genus *Malassezia* (class Malasseziomycetes, subphylum Ustilaginomycotina, phylum Basidiomycota) comprises a group of lipid-dependent yeasts that are part of the human population skin mycobiome [1]. Though they are typically associated with common skin disorders, they are increasingly recognized as causes of invasive infections in children. Three species of *Malassezia*, *Malassezia furfur*, *Malassezia pachydermatis*, and *Malassezia sympodalis,* have been implicated in invasive infections found in humans [2].

*M. pachydermatis* is a zoophilic species that is part of the normal skin flora of dogs and cats [3] but has been implicated in human cases of catheter-related bloodstream infection, meningitis, and death [2, 4–6]. Pediatric cases are almost exclusively reported in premature infants on parenteral nutrition or immunocompromised children [2]. We describe an unusual case of invasive *M. pachydermatis* infection in a patient who is demographically unique compared with other pediatric cases.

## 2. Case Description

An 8-year-old girl with a history of short gut syndrome secondary to gastroschisis presented with fevers and fatigue. She received enteral feeds but was dependent on parenteral lipid nutrition (provided by the four-oil emulsion Smoflipid^®^) through a central venous catheter (CVC). She denied any new rashes, skin changes, or gastrointestinal symptoms including abdominal pain, diarrhea, or intolerance to enteral feeds. Her exposure history was notable for a 6-year-old pitbull at home.

**Figure 1:**
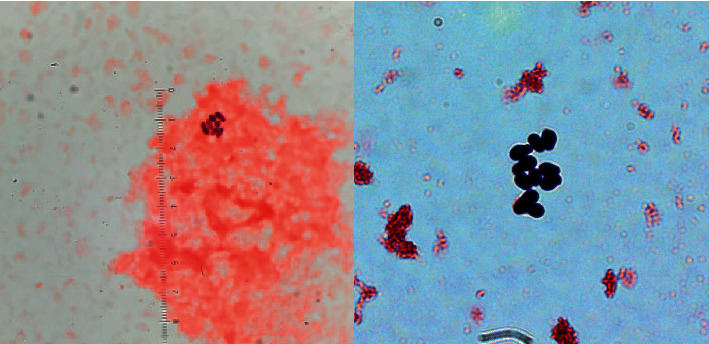
Gram-stained smear of positive blood culture revealing yeast-like cells with collarettes suspicious for a non-*Candida* organism, 40*x* and 100*x*.

On presentation, she was febrile to 100.6°F, but her remaining vital signs were within normal limits for her age. Her physical examination, including inspection of her CVC site, was unremarkable. The results of a complete blood count and C-reactive protein were within normal limits. Blood cultures (BacT/Alert^®^) obtained from the CVC subsequently flagged positive after 28 hours of incubation. A direct Gram-stained smear revealed yeast-like cells (Figure 1) that were approximately 3 × 5 *μ*m with collarettes, making *Candida* species unlikely.

**Figure 2:**
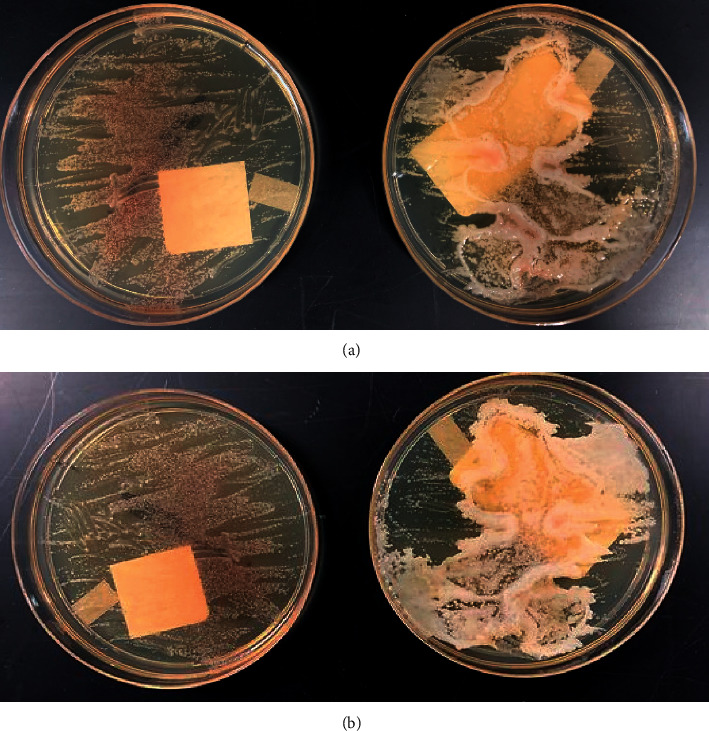
Growth on SDA vs. SDA with long-chain fatty acid supplementation, 30°C. Day 5 (a) vs. day 10 (b).

Slow growth was demonstrated on Sabouraud-dextrose agar (SDA) with and without long-chain fatty acid supplementation although more robust growth occurred with fatty acid supplementation over time (Figure 2).

The isolate was subsequently identified as *M. pachydermatis* using mass spectrometry time of flight (MALDI-TOF) (BioMerieuxVitek® MS) with 99.9% confidence.

The patient was started on intravenous fluconazole (12 mg/kg/day) when the Gram-stained smear was reported, and an Infectious Diseases consultation was sought upon organism identification. The primary team attempted a line salvage strategy with intravenous antifungals alone as her nutritional requirements prevented discontinuation of parenteral lipid nutrition and instillation of antifungal locks. However, growth of yeast was documented on daily blood cultures obtained from the CVC for the following 5 days. Transthoracic echocardiogram and ophthalmologic funduscopic exam did not reveal evidence of cardiac or ocular involvement.

The CVC was ultimately removed and replaced with a peripherally inserted central catheter (PICC) on day 8, after which she had no further positive cultures. Results of antifungal susceptibility testing results became available on day 12, and based on these results, we decided to replace intravenous fluconazole (minimal inhibitory concentration (MIC) = 16 *μ*g/ml) with intravenous posaconazole at 10 mg/kg daily (MIC <0.03 *μ*g/ml). Voriconazole was another alternative option based on susceptibility testing (MIC 0.125 *μ*g/ml). It was planned to continue therapy for 14 days, but the patient completed 18 days of antifungals before the reinsertion of a new CVC, after which she was discharged home.

## 3. Discussion

The majority of case reports describing invasive disease due to *M. pachydermatis* involved neonates on parenteral nutrition or immunocompromised children [2]. Our patient was demographically distinct from these children and was an unexpected case of invasive disease due to *M. pachydermatis*.

The major risk factor contributing to our patient's infection was likely the receipt of parenteral lipid nutrition and the use of a CVC. Parenteral lipid nutrition is commonly reported as a risk factor, mainly in neonatal infections, due to the lipid dependence of *Malassezia* species [2]. *M. pachydermatis* was traditionally considered the only species within the genus not to be strictly lipid dependent [1, 6]. This idea came from the observation that *M. pachydermatis* can grow on SDA without fatty acid supplementation. However, *M. pachydermatis* was recently discovered to lack the gene encoding for fatty acid synthase, as with all other *Malassezia* species. Its growth on nonsupplemented SDA was explained by the discovery that commercial peptone used to prepare SDA contains small amounts of fatty acids [1]. In a completely lipid-free media, the growth of *M. pachydermatis* does not occur.

The dog exposure reported by our patient is worth discussing as *M. pachydermatis* is a known animal skin commensal with zoonotic potential given that dog owners are at increased rates of *M. pachydermatis* carriage [7]. During an outbreak investigation of 15 cases of *M. pachydermatis* in a neonatal intensive care unit, *M. pachydermatis* was also isolated from the skin of a healthcare worker and several dogs owned by different healthcare workers [4]. These isolates had similar genetic signatures based on genotyping studies, implicating dog-owning healthcare workers as potential vectors that allowed colonization of infants with *M. pachydermatis.* In our case, there were no cultures performed from the patient's dog to prove this association. Still, we suspect that the contact with the dog gave origin to the infection.

This case also illustrates the challenges with diagnosis and management of disease due to *Malassezia* species including *M. pachydermatis*. Cases of infection caused by *Malassezia* species may be underdetected because fatty acid supplementation is not routinely performed when culturing yeast on solid media. Fatty acid supplementation is also important for *M. pachydermatis* which can grow on some commercial lipid-free media, but the growth is more vigorous in the presence of fatty acids. Supplementation should be considered, especially if Gram-stain smears indicate the presence of yeast-like cells but growth is slow or lacking on solid media, as seen in our case.

Treatment of infections with *Malassezia* species is guided primarily by anecdotal experience in the absence of comparative studies and lack of standardized susceptibility testing [8, 9]. Amphotericin B or fluconazole are commonly utilized as empiric therapy [8] although in vitro resistance to fluconazole has been reported, especially for *M. pachydermatis* [10–13]. In regards to our case, we replaced fluconazole with posaconazole after susceptibility test results were made available, though at that time, the infection appeared under control. The switch to another antifungal agent was made prudentially since the very low MIC suggested a higher effectiveness of posaconazole. The apparent discrepancy between the high MIC and clinical effectiveness of fluconazole may be explained by the high dosage employed, which may have allowed reaching therapeutic blood levels. Another explanation is that the removal of the CVC contributed to the success of the fluconazole treatment.

In addition to removal of CVCs, other key aspects in managing *Malassezia* fungemia include reducing or eliminating lipid parenteral nutrition [8, 14]. Although CVC salvage may be desired, *Malassezia* species are known to form biofilms in a capacity similar to *Candida* species which may make this strategy ineffective [15, 16]. The efficacy of antifungal lock therapy for salvage of CVC-associated infections is unknown, but its successful use has been reported in one instance [17]. Not surprisingly, our patient failed catheter salvage with persistent growth of *M. pachydermatis* despite a week of antifungal therapy.

In conclusion, invasive disease due to *M. pachydermatis* is an uncommon, opportunistic infection and should also be considered for children on lipid parenteral nutrition irrespective of age or immune status. The incidence of invasive *M. pachydermatis* in older, immunocompetent children is likely underrecognized, and clear communication of a suspicion or risk of any *Malassezia* species can help the microbiology laboratory consider fatty acid supplementation of solid media culture.

## Data Availability

All underlying data used to support the findings of this study are included within the article.
